# Content and comprehensiveness in the nursing documentation for residents in long-term dementia care: a retrospective chart review

**DOI:** 10.1186/s12912-022-00863-9

**Published:** 2022-04-11

**Authors:** Lene Baagøe Laukvik, Merete Lyngstad, Ann Kristin Rotegård, Åshild Slettebø, Mariann Fossum

**Affiliations:** 1 Department of Health and Nursing Science, Faculty of Health and Sport Sciences, University of Agder, PO Box 509, NO-4898 Grimstad, Norway; 2 Norwegian Nurses Organization, Oslo, Norway; 3 VAR Healthcare, Cappelen Damm, Oslo, Norway

**Keywords:** Clinical audit, Dementia, Long-term-care, Nursing care, Patient participation

## Abstract

**Background:**

Insight into and understanding of content and comprehensiveness in nursing documentation is important to secure continuity and high-quality care planning in long-term dementia care. The accuracy of nursing documentation is vital in areas where residents have difficulties in communicating needs and preferences. This study described the content and comprehensiveness of nursing documentation for residents living with dementia in nursing homes.

**Methods:**

We used a retrospective chart review to describe content and comprehensiveness in the nursing documentation. Person-centered content related to identity, comfort, inclusion, attachment, and occupation was identified, using an extraction tool derived from person-centered care literature. The five-point Comprehensiveness in the Nursing Documentation scale was used to describe the comprehensiveness of the nursing documentation in relation to the nursing process.

**Results:**

The residents’ life stories were identified in 16% of the reviewed records. There were variations in the identified nursing diagnoses related to person-centered information, across all the five categories. There were variations in comprehensiveness within all five categories, and inclusion and occupation had the least comprehensive information.

**Conclusion:**

Findings from this study highlights challenges in documenting person-centered information in a comprehensive way. To improve nursing documentation of residents living with dementia in nursing homes, nurses need to include residents’ perspectives and experiences in their planning and evaluation of care.

**Supplementary Information:**

The online version contains supplementary material available at 10.1186/s12912-022-00863-9.

## Background

According to the World Health Organization, approximately 55 million people worldwide are currently physically, psychologically, socially, and economically impacted by dementia, and this number is expected to increase [1]. Dementia is characterized as a progressive chronic neurocognitive disease that impacts one or more cognitive domains, causing loss of verbal abilities and resulting in a complete dependence in activities of daily living [2]. International research shows that more than half of nursing home residents suffer from dementia [3]. In Norway, estimates show that nearly 80% of residents living in nursing homes have some form of dementia and that around 25% of nursing homes are part of special care units for people with dementia [4]. The healthcare workforce in nursing homes consists of both professionals and nonprofessionals with a mix of nurses and nurse aides involved in daily care planning and documentation of nursing care in the electronic health records (EHRs) of residents living with dementia [5, 6]. Access to accurate and reliable information in the EHRs of residents living with dementia is important to secure continuity, quality, and safety of the residents [7, 8]. The global action plan on the public health response to dementia [1], emphasizes that the sharing of high-quality data relevant to dementia care is important to improve the healthcare trajectories of persons suffering from dementia.

Person-centered care (PCC), is increasingly considered as high-quality care in dementia long-term facilities, wherein individualized care planning, informed by the residents’ history, needs, and preferences, are recommended [9, 10]. PCC is an important part of the culture change movement and is highly profiled in long-term care for older adults in the 2018 Alzheimer’s Association Demementia Care Practice Recommendations [11]. Recent research shows that individualized and personalized information about the resident can improve nurses’ knowledge and attitudes and adjust care-delivery accordingly, in dementia care [12, 13].

The concept of PCC is not defined unambiguously, and several PCC models exist in the literature [10]. In this study, we employ the work of Kitwood [14–16], to help clarify the concept of PCC. Basic needs identified by Kitwood and Bredin [17], such as identity, comfort, inclusion, attachment, and occupation, are considered as particularly important to comprehensively document to ensure high-quality care planning, in the context of dementia care. *Identity* reflects the need to know who you are, both cognitively and emotionally, and to have a sense of continuity with the past [15]. *Comfort* reflects the need for warmth and strength from other people, which can enable the resident to remain peaceful when they are in danger of deterioration. *Inclusion* reflects the need to be involved in, and to maintain relationships and a social life. *Attachment* reflects the need to establish and experience emotional bonds. *Occupation* reflects the need to be involved in life processes, in a way that is personally significant [15]. Learning about the resident’s life story is a key indicator for understanding who the resident is, which needs are prominent, and what is the best way to approach these needs [11, 12]. Recognizing and maintaining selfhood is key to PCC. Through the written life story, nurses and other members of the healthcare team can learn about the social context of the resident’s life, roles, values, relationships, losses, and sense of self, and use this information in the development of person-centered care plans [11, 13]. Recent research shows that documentation of the life stories of residents in nursing homes can improve communication and the quality of relationships between the residents, their relatives, and healthcare professionals significantly [18]. However, the actual use of life stories in clinical practice varies across healthcare settings [19, 20].

Quality care planning and documentation of nursing in dementia care is a complex ongoing process that must reflect the unique needs and experiences of the resident [5, 21]. Nursing home residents living with dementia often cannot articulate their needs and preferences, and adequate and comprehensive information enables nurses to meet the basic needs of the residents and promotes their well-being [6, 12]. Comprehensiveness in nursing documentation is defined as documentation according to the nursing process using an unambigous language [7, 22]. The nursing process model, consisting of assessment, diagnosis, planning, implementation, and evaluation is implemented as the basic structure to record nursing care in several EHR systems [22, 23]. Sufficient documentation of the core elements in the nursing process may enable nurses in long-term facilities to obtain a more complete picture of the residents and adjust care delivery accordingly [24, 25]. Standarized nursing language (SNL) to describe nursing care is developed and implemented to support nurses in documenting accurate and comprehensive information, which shows a positive effect on the structure and descriptions of the elements of the nursing process[26]. In Norway, the Norwegian Directorate of eHealth [27] recommends the use of the International Classification for Nursing Practice (ICNP) for nursing documentation in clinical practice. However, the implementation of SNL is in its early stages and has been done partially in Norway [28]. Despite development of quality critera and positive support for nursing documentation, research in both community and hospital care, show inaccuracies in the recorded content, such as insufficient and incomplete documentation of the nursing process-elements [22, 24, 29], and a lack of recorded person-centered information [30, 31]. Lack of adequate content and comprehensiveness in nursing documentation may cause potential misunderstandings and misintrepretations, thus jeopardising the safety of residents [6, 7].

An insight into the content of recorded nursing care of residents living with dementia could provide knowledge of how to focus their basic needs in the care planning and documentation of nursing to preserve a sense of personhood in daily living [11, 12]. Knowledge and insight into the content and comprehensiveness of the recorded information of such nursing care, may help nurses to better understand how to effectively communicate comprehensive individual and person-centered information, to facilitate continuity of high-quality care [6, 7]. Thus, this study aimed to describe the content and comprehensiveness of nursing documentation for residents living with dementia in nursing homes in relation to identity, comfort, inclusion, attachment, and occupation.

## Methods

A cross-sectional retrospective chart review of resident records was conducted, to describe the content and comprehensiveness of nursing documentation for residents living with dementia in nursing homes, in relation to the following PCC-themes: identity, comfort, inclusion, attachment, and occupation [32]. An auditing instrument: Comprehensiveness In Nursing Documentation, CIND, was used to evaluate the comprehensiveness of the nursing documentation [33]. Data was analyzed using descriptive statistics and summarized in tables and figures [34]. The Strengthening the Reporting of Observational studies in Epidemiology (STROBE) checklist was used to ensure quality reporting (Additional file 1).

### Sample and setting

A constructive sample of residents [34], currently living in long-term dementia care units in two large (populations of 40,000–50,000) municipalities and one medium (population of 19,000) municipality were recruited for this study. The residents or their next of kin were approached by the nurse unit manager who provided oral and written information about the study. Out of a total of 173 identified eligible residents, 121 agreed to participate, allowing their records to be audited for research purposes. The inclusion criteria were: (a) all residents currently living in special care units for older adults at (b) a public nursing home that (c) had access to the EHR system that supported documentation of nursing care according to the nursing process model. According to local guidelines, in all study sites, registered nurses and assistant nurses were responsible for the development of NCPs. However, there was no official routine as to when the the plans were to be updated. In all the study sites, all staff, with and without special education in dementia care, and nursing aides without any healthcare education, had access to write daily reports/progress notes (PNs) in the EHR. The EHR system in all study sites was structured according to the nursing process model, with freewriting for nursing diagnoses, resident outcomes, and interventions in the NCPs, and evaluation of outcomes in the PNs. Assessment charts and the life story of the resident were documented in separate files. Nursing documentation in this study constitutes the life story of the resident, assessment charts, NCPs, and PNs.

### Data collection

Data were collected between January 2019 and April 2019. Retrospective data from three months prior were extracted from the residents’ records. This method has been found to be effective in previous studies [35]. Documentation from other institutions and physician reports were not reviewed. One of the researchers was present at the nursing home during the printing process to support this work, if needed. Information on residents’ gender, age, and length of stay was collected from the EHR, in addition to the nursing documentation.

### Instrument

The comprehensiveness in the nursing documentation was reviewed for specific problems related to identity, comfort, inclusion, attachment, and occupation, using an instrument developed by Ehnfors and Smeby [33], the comprehensiveness in nursing documentation scale, CIND. Comprehensiveness in this study was defined as whether the information was recorded in accordance with the nursing process. Specific descriptions of the CIND scores are presented in Table 1. The total score ranges from 1 to 5, where 5 indicates the most comprehensive documentation, which includes: (a) a recorded nursing diagnosis (ND), (b) planned and implemented nursing interventions, (c) recorded nursing outcomes, and (d) a recorded evaluation of the steps in the nursing process. A guide, based on PCC-literature, was used for sorting recorded content into the themes of identity, comfort, inclusion, attachment, and occupation [32].

**Table 1:** Comprehensiveness scores according to CIND^a^ and corresponding descriptions of the PCC^b^-categories in the nursing documentation

Grading protocol for the comprehensiveness-score		Description of scores	Example of scores with statements from the nursing care plan and progress notes
Score of 1	The problem is described, *or* interventions planned *or* implemented	[1] PCC-ND^c^ **or** [2] PCC-NIV^d^ is present in NCP^e^, **or** [3] statement that the PCC-NIV is completed	[1] “Needs assistance in managing own behavior” **or** [2] “One-on-one follow-up when signs of agitation” **or** [3] “The resident has been agitated today. They received one-on-one follow-up.”
Score of 2	The problem is described, *and* interventions planned *or* implemented	[1] PCC-ND **and** [2] PCC-NIV is present in NCP, **or** [3] statement that the PCC-NIV is completed	[1] “Needs assistance in managing own behavior” **and** [2] “One-on-one follow-up when signs of agitation” **or** [3] “The resident has been agitated today. They received one-on-one follow-up.”
Score of 3	The problem is described, *and* intervention planned *or* implemented, *and* resident outcome is recorded	[1] PCC-ND **and** [2] PCC-NIV are present in NCP, **or** [3] statement that PCC-NIV is completed **and** [4] PCC-NO^f^ is recorded	[1] ‘Needs assistance in managing own behavior” **and** [2] “One-on-one follow-up when signs of agitation” **or** [3] “The resident has been agitated today. They received one-on-one follow-up **and** [4] without any effect.”
Score of 4	The problem is described, *and* intervention planned *and* implemented, *and* nursing outcome is recorded	[1] PCC-ND **and** [2] PCC-NIV are present **and** statement [3] that the PCC-NIV is completed **and** [3] PCC-NO is recorded	[1] “Needs assistance in managing own behavior” **and** [2] “One-on-one follow-up when signs of agitation” **and** [3]“The resident has been agitated today. They received one-on-one follow-up **and** [4] without any effect.”
Score of 5	All aspects of the nursing process are recorded, including nursing history, diagnosis, goals, and discharge notes. There is an adequate description of the problem. The recording is of relevance to nursing	[1] PCC-ND described adequately with present [2] PCC-NG^g^ and [3] PCC-NIV is present. Statement that the [4] PCC-NIV is completed [5], PCC-NO is recorded, an evaluation of the resident’s experience of completed PCC-NIV is recorded	[1] “Needs assistance in managing own behavior. The resident has no insight into own health condition. Reacts negatively with anger to changes made to surroundings. Has reacted in a hostile manner toward other residents, relatives and staff.” [2] “Resident feels seen and understood.” [3] “One-on-one follow-up when signs of agitation occur.” [4] “The resident has been agitated today. They received one-on-one follow-up without any effect. [5] They were in despair and started crying when expressing how much they missed their partner.”

^a^*CIND* Comprehensiveness in nursing documentation

^b^*PCC* Person-centered care;

^c^*ND* Nursing diagnosis

^d^*NIV* Nursing intervention

^e^*NCP* Nursing care plan

^f^*NO* Nursing outcome

^g^*NG* Nursing goal

### Procedure and data analysis

The sample size of records was based on estimates drawn from similar study designs [29, 36]. First, whether the life story was registered in the records (yes/no) was recorded. Then, whether the assessment notes were registered in the records, and if the identified notes contained information relating to identity, comfort, inclusion, attachment, and occupation, following the guide (yes/no), was recorded. Thereafter, free-text-written nursing diagnoses in relation to psychosocial needs were identified in the NCP and organized into identity, comfort, inclusion, attachment, and occupation, following a guide (Additional file 2). Next, information connected to the identified ND was tracked throughout the NCP and PNs; subsequently given a score of 1–5 for comprehensiveness (Table 1). All scores were transferred to SPSS version 25 (IBM Corp., Armonk, NY, USA), and analyzed using descriptive statistics [34].

A modified form of the nominal group technique (NGT) was used in the validation process of CIND and the extraction tool [37]. The reviewers (LBL and MF) involved in the validation process were familiar with the chart review technique, and they had knowledge and experience in dementia care in long-term care settings, and in documenting nursing care. First, one author (LBL) identified and sorted NDs according to the categories and evaluated the comprehensiveness of the information using CIND and the extraction tool from two records for training purposes. Then, two members of the research group (LBL and MF) individually evaluated five new records using CIND and the extraction tool. The evaluations were compared and discussed face-to-face until an acceptable agreement was reached. The decision rules were created (Table 2). Second, the same authors (LBL and MF) individually evaluated 12 new records using CIND and the extraction tool. The evaluations were compared and discussed face-to-face until an acceptable agreement was reached. All records used for training purposes and discussions (*N* = 19) were audited again by the first author and included in the data analysis.

**Table 2:** Number of decision rules for data coding and extraction

1. No specific structure required for the nursing diagnose formulation for selection from the nursing care plan
2. The interventions present in the nursing care plan must be connected or related to the identified nursing diagnoses
3. All recorded interventions (connected to the identified nursing diagnoses) are implemented when it is recorded that the intervention has been done (completed)
4. Recorded nursing goals and nursing outcomes need to be clearly formulated as resident outcomes

## Results

A total of 173 eligible residents’ records were identified, and 121 residents from a total of 21 special care units in seven public nursing homes agreed to participate in this study, allowing their records to be audited for research purposes. The mean age of the residents was 84 years (standard deviation = 8, range = 64–100), and 87 residents (71.9%) were women. The mean length of a nursing home stay was 28 months (standard deviation = 25.7, range = 1–100).

### Person-centered content

The life story of the residents was identified in only 19 (15.7%) of the reviewed records. Assessment charts, containing information relevant for the PCC categories, were identified in 100 (82.6%) of the records. NCPs containing nursing diagnoses (NDs) related to identity, comfort, inclusion, attachment, and occupation were identified in 104 (86%) records (Table 3).

**Table 3:** Number of records containing PCC^a^-information in the reviewed records (*n* = 121)

Content identified	Frequency	Percent
Life story	19	15.7
Assessment charts containing PCC^a^ relevant information	100	82.6
Nursing care plan	121	100
Nursing diagnoses related to the PCC-categories in nursing care plan	104	86.0

^a^Person Centered Care

Within the 104 records containing PCC-related NDs, a total of 372 (mean 4, range 1 -8) NDs were identified. One hundred and twenty-nine (35%) of the NDs contained content related to identity and 27 (7%) contained information related to inclusion. Table 4 shows the total number of identified NDs in each PCC category. The identified NDs were most often brief statements about the resident’s general condition. The content of the NDs were most commonly related to pain, behaviour, activity and family matters, e.g. “The resident has a headache”, “The resident is restless,” “Need for activity” and “Contact with family,” without any proper description of signs and symptoms. All identified NDs, goals, and planned interventions in the NCP were written as free texts. Evaluations and resident outcomes were written as unstructured free text in the PNs in all the 104 records.

**Table 4:** CIND^a^ score within the PCC^b^-categories based on identified NDs^c^ (*N* = 372). Total number of records: 104

	**CIND**^**a**^** score**					Total NDs^c^
	1	2	3	4	5	
	The problem is described, **or** intervention planned **or** implemented	The problem is described, **and** intervention planned **or** implemented	The problem is described, **and** intervention planned **or** implemented, **and** nursing outcome is recorded	The problem is described, **and** intervention planned **and** implemented, **and** nursing outcome is recorded	All aspects of the nursing process are recoded. Good description of the problem and recoding of the relevance for nursing	
PCC^b^ category	*f* (%)	*f* (%)	*f* (%)	*f* (%)	*f* (%)	*f* (%)
Identity	14 (3.8)	34 (9.2)	44 (11.9)	36 (9.7)	1 (0.3)	129 (34.7)
Comfort	9 (2.4)	28 (7.6)	13 (3.5)	32 (8.6)	0	82 (22.0)
Inclusion	3 (0.8)	12 (3.2)	3 (0.8)	8 (2.2)	1 (0.3)	27 (7.3)
Attachment	6 (1.6)	16 (4.3)	20 (5.4)	24 (6.5)	1 (0.3)	67 (18.0)
Occupation	4 (1.1)	17 (4.6)	32 (8.6)	14 (3.8)	0	67 (18.0)
Total *f*(%)	36 (9.7)	107 (28.8)	112 (30.1)	114 (30.7)	3 (0.8)	372

^a^*CIND* Comprehensiveness in nursing documentation

^b^*PCC* Person centered care

^c^*NDs* Nursing diagnoses

### Comprehensiveness

A CIND score of 4 was achieved with 114 (31%) of the 372 identified NDs across all PCC categories, meaning that the resident’s problem and corresponding planned interventions were stated, and an effect of the implemented interventions were stated. Only three (1%) of 372 identified NDs achieved a CIND score of 5, meaning that all aspects of the nursing process were recorded, containing descriptions of the resident’s experience. Table 4 shows the distribution of the scores achieved in accordance with the PCC variables.

## Discussion

The findings of this study highlight issues of nursing documentation important for the planning and implementation of PCC in long-term dementia care. The lack of documentation of the residents’ life stories found in this study could indicate that the EHR system used for nursing documentation had limitations concering structures and content for recording the life stories. Recent research shows that the provision of appropriate structures or templates in the EHR system for facilitating recording of background information enables nurses in the documentation of individualized and personalized information [6]. Unique information about the defining moments in the residents’ lives should be registered and provided as a whole in the nursing documentation in order for nurses to relate and interact with the resident in a way that is meaningful and safe [13].

Earlier research suggests that a poor standard of life story records in care planning and nursing documentation could be a result of the motivations behind writing or creating these stories [18, 19]. However, several studies show that nurses and other healthcare professionals have positive attitudes towards using life stories for quality care planning and delivery of care for residents living with dementia [12, 20, 38]. On the other hand, life stories might contain sensitive information, causing an avoidance of recording such information due to the ethical aspects of resident participation in writing their life stories [19]. If the values and beliefs of the resident are not reflected in the nursing documentation, it could hinder nurses and other members of the healthcare team in accommodating the residents’ individual daily routines, learn about who the residents are, and provide all residents with a variety of activities [11].

Even if the number of registered life stories were low in this study, some of the recorded interventions in care plans, especially related to activity, were based on what the resident had previously enjoyed, such as “went to church every Sunday with their partner” or “used to work on a farm all their adult life.” Such information in the nursing documentation can contribute to the creation of a proactive care plan that responds to the behavioral and psychosocial symptoms of dementia [12, 19].

This study found variations between the number of identified assessment charts containing PCC-relevant information and the number of NCPs containing PCC-related NDs. In addition, the NDs were commonly lacking in descriptions of what led to the NDs. This could imply that relevant assessment-data was not used in forming and deciding NDs in the care planning process. Similar problems have been identified by Tuinman, de Greef [24], and Wang, Yu [25], in study settings where NDs were required. A disconnection between information about contributing factors that lead to the stated ND can create serious gaps in the nursing documentation. Such gaps can create interpretations and assumptions of relevant needs and desires that could threaten individualized care planning and the safety of the resident [10, 19]. However, some of the NDs identified in this study contained descriptive information about what led to the ND; typically an observation of the resident’s behavior or emotions (see example in Table 1). If descriptions of contributing factors connected to the stated ND are provided, they might facilitate better understanding of the nature of the identified problem. This could stimulate nurses’ engagement in the clinical reasoning process of deriving a sound and clinically meaningful ND, as the basis for further care planning [22, 25]. Structured documentation that demonstrates how the condition of the residents living with dementia has been understood can contribute to ensuring that they are valued and respected as persons [11]. By connecting information about signs and symptoms that led to the NDs into the NCP, nurses in dementia care can identify and implement appropriate interventions to achieve desired person-centered outcomes [22, 39].

A comprehensive recording of the nursing process containing an evaluation of care based on the residents’ perspectives and experiences were only found in three NDs in this study. An explanation for this low number might be related to challenges in expressing and formulating personalized information during documentation [19]. Previous studies suggest that information concerning physical aspects of care are more familiar to nurses, resulting in a more distant and objective language in the nursing documentation, making PCC-planning difficult [40, 41]. The implementation and use of SNL related to psychosocial information could increase the comprehensiveness and person-centeredness in the nursing documentation investigated in this study [7, 23].

When comprehensiveness was high in this study, the documentation included information about the residents’ expressed feelings and/or nurses’ observed response to care indicating that the residents’ descriptions of their own situation and response to care should form the content of evaluations of nursing care [12]. Increased focus on the perspectives and experiences of the resident in care planning, and documentation of nursing for residents living with dementia, can create an environment in the nursing home that respects and maintains the selfhood of the resident [11].

### Strength and limitations

One of the strengths of this study is that it provides valuable information about documentation of nursing care to residents living with dementia in long-term care. Our findings do not represent the content and comprehensiveness of all long-term residents suffering from dementia; therefore, they cannot be generalised. However, the findings represent care planning and documentation of nursing in the context of dementia care in nursing homes. The extraction tool used in this study may not have been conceptually and visually clear enough to avoid errors in the identification of content in relation to the PCC-categories. An extraction tool was provided with a description and examples of themes derived from established PCC-literature, to help the reviewer identify appropriate statements. To minimise the subjective factors in the identification and coding of data, training and validation processes was completed through thorough discussions among members of the research team [32, 37].

## Conclusions

Findings from this study show challenges in documenting person-centered information in a comprehensive way. Serious flaws in the nursing documentation of residents living with dementia, such as incomplete documentation of the steps in the nursing process and lack of registered life story can create assumptions and interpretations jeopardising the safety of the residents. To improve nursing documentation of residents living with dementia in nursing homes, nurses need to include residents’ perspectives and experiences in their planning and evaluation of care. Failure to comprehensively record information related to psychosocial aspects can make it impossible to understand whether important basic needs have been considered in the evaluation processes or whether interventions are appropriate and should be continued. Further qualitative research should be conducted to obtain an in-depth insight into nurses’ attitude toward PCC and the documentation process, including the use of terminology related to psychosocial needs. Such insight could help further understand how to comprehensively document nursing care of residents living with dementia.

## Supplementary Information

**Additional file 1**.

**Additional file 2**.

## Data Availability

The datasets used and/or analyzed during the current study are available from the corresponding author on reasonable request.

## Abbreviations

CIND: Comprehensiveness in nursing documentation
EHR: Electronic health records
ICNP: International classification for nursing practice
NCP: Nursing care plan
NDs: Nursing diagnosis
NG: Nursing goal
NIVs: Nursing interventions
NO: Nursing outcome
PCC: Person-centered care
SNL: Standardized nursing language
