# Supplementary material for: Content and comprehensiveness in the nursing documentation for residents in long-term dementia care: a retrospective chart review
Source: BMC Nurs. 2022 Apr 11;21:84. doi: 10.1186/s12912-022-00863-9 (PMC9004102; doi:10.1186/s12912-022-00863-9)
Supplement: Supplementary file 2 — Additional file 2. [file 12912_2022_863_MOESM2_ESM.docx]

# Data extraction guide for Person Centred Care (PCC) content, derived from PCC literature

| **Category**  **(code)** | **Theoretical definition** | **Questions for identification of appropriate statements to code**  **Yes/no**  **Where found? (NCP or PN or both)** | **Examples of theme in statements**  **(maximum three statements for each question should be extracted for examples)** |
| --- | --- | --- | --- |
| **Identity** | To know who you are  Your own (and others’) experiences  Cognitive and emotional  Autonomy  To some degree our identity is defined by other people through the subtle messages they express about our performance. | Resident’s communication of own needs/preferences? | Resident’s expression of needs, what he/she wants/desires, pain, sleep, hungry, wants to go home |
|  |  | Resident’s needs associated with emotions? | Statements about the resident’s feelings (observed). F.ex: happy, sad, angry, indifferent |
|  |  | Resident’s needs associated with behaviour? | About the resident’s behaviour (observed). F.ex: wandering, restless, aggressive, and calm |
|  |  | Resident's experience of own behaviour? | Expressions describing how the resident experiences his/her behaviour in a situation |
|  |  | Resident’s needs associated with cognition? | Statements about the resident’s cognition (observed). F.ex: disoriented, memory loss |
|  |  | Resident’s experience of own condition/illness? | Expressions describing resident’s experience of his/her condition or illness, situation in life or the nursing home |
| **Comfort** | Proximity, tenderness, relief from pain and grief, reassurance to relieve anxiety and grief.  The feeling of security that comes from being close to another person.  Maintaining a wholeness when you experience falling apart together (in parts, subdivided).  The need is strongest in managing loss. | Resident’s experience of well-being? | Statements about the resident’s experience of pain or other discomfort. Statements about planned care connected to pain, expressed as “pain” in the nursing care plan.  Statements about mental state |
|  |  | Resident’s experience of his/her own emotions? | Expressions describing resident’s experience related to his/her emotions |
|  |  | The quality of interaction between resident and staff? | The quality of the interaction expressed, see the interaction from the resident’s perspective |
| **Inclusion** | Social dimension  Being part of a group    Primarily, being part of a group is essential for human survival.  In dementia, this need arises especially through attention-seeking behaviour. | Use of non-verbal communication interaction? | Sitting silently with the patient, changing staff members, providing diversion silently |
|  |  | Resident’s experiences of communication with others? | Family, friends, relatives, volunteers, other residents, staff |
|  |  | Facilitation of the environment to safeguard the resident’s autonomy? | Facilitate autonomy in everyday situations (grooming, meals, social activities, and navigation) |
|  |  | Use of coercion in interaction between resident and staff? | Locked doors  Physical restraint, holding patient down, remaining in room |
| **Attachment** | Social nature, attachment, and bonding  Establishing emotional bonding, connecting with others is instinctive.  Difficult for a person to function without having a sense of belonging. Loss of primary attachment can have a negative impact. | Facilitating a home-like environment for the resident? | Resident room or common area  Use of biography: pictures, conversations about “old times” (family, relatives, friends, work, interests), life history, religion |
|  |  | Needs related to social relationships? | Family, friends, relatives, volunteers visiting, other residents. Statements of social relations, documented visits |
|  |  | Resident’s experience of/with social relationships? | Description of the resident’s experience of the abovementioned relationships |
| **Occupation** | Being involved. The opposite is boredom, apathy, feeling useless  Being involved in the life process in a way that is personally significant.  Draws on a person's abilities and strengths. | Participation in activities? | If the resident has participated in any social or physical activity |
|  |  | Needs associated with activities? | What needs does the resident have related to social/physical activity? |
|  |  | Patient experience of participation in activities? | Expressions describing the resident’s experience of participating in an activity |
|  |  | Resident’s use of the outdoor area? | If the patient uses the outdoor facilities or organized outdoor excursions |

**References:**

Edvardsson, Winblad & Sandman, 2008. Person-centred care of people with severe Alzheimer’s disease: current status and ways forward*. The Lancet, 7*(4), 362-367. Doi: https://doi.org/10.1016/S1474-4422(08)70063-2

Brooker, D. (2003). What is person-centred care in dementia? *Reviews in Clinical Gerontology, 13*(3), 215-222. Retrieved from <http://search.proquest.com/docview/211549769?accountid=45259>. doi: 10.1017/S095925980400108X

Brooker, D., & Latham, I. (2019). *Person-Centred Dementia Care* (Second Edition ed.). London, UK and Phladelphia, USA: Jessica Kingsley *Publishers*.

Kitwood, T. (1993). Towards a Theory of Dementia Care: The Interpersonal Process. *Ageing and Society, 13*(01), 51-67. doi:10.1017/S0144686X00000647

Kitwood, T. (1997). The experience of dementia. *Aging & Mental Healt, 1:1*, 13-22. doi: 10.1080/13607869757344

Kitwood, T. (2019). *Dementia Reconsidered, Revisited - The Person Still Comes First* (D. Brooker Ed. 2. ed.): Open University Press.

Shiells, K., Holmerova, I., Steffl, M., & Stepankova, O. (2019). Electronic patient records as a tool to facilitate care provision in nursing homes: an integrative review. *Informatics for Health & Social Care, 44*(3), 262-277. doi:10.1080/17538157.2018.1496091

Stein-Parbury, J., Chenoweth, L., Jeon, Y. H., Brodaty, H., Haas, M., & Norman, R. (2012). Implementing Person-Centered Care in Residential Dementia Care. *Clinical Gerontologist, 35*(5), 404-424 421p. doi:10.1080/07317115.2012.702654

Teitelman, J., Raber, C., & Watts, J. (2010). The power of the social environment in motivating persons with dementia to engage in occupation: qualitative findings. *Physical & Occupational Therapy in Geriatrics, 28*(4), 321-333. doi:10.3109/02703181.2010.532582
